# Bioinformatics Analysis of Key Genes and circRNA-miRNA-mRNA Regulatory Network in Gastric Cancer

**DOI:** 10.1155/2020/2862701

**Published:** 2020-08-22

**Authors:** Yiting Tian, Yang Xing, Zheng Zhang, Rui Peng, Luyu Zhang, Yan Sun

**Affiliations:** ^1^Department of Cell Biology and Genetics, Chongqing Medical University, Chongqing 400016, China; ^2^Clinical Laboratory, Chongqing University Central Hospital, Chongqing 400014, China; ^3^Department of Bioinformatics, Chongqing Medical University, Chongqing 400016, China

## Abstract

Gastric cancer (GC) is one of the most common malignancies in the world, with morbidity and mortality ranking second among all cancers. Accumulating evidences indicate that circular RNAs (circRNAs) are closely correlated with tumorigenesis. However, the mechanisms of circRNAs still remain unclear. This study is aimed at determining hub genes and circRNAs and analyzing their potential biological functions in GC. Expression profiles of mRNAs and circRNAs were downloaded from the Gene Expression Omnibus (GEO) data sets of GC and paracancer tissues. Differentially expressed genes (DEGs) and differentially expressed circRNAs (DE-circRNAs) were identified. The target miRNAs of DE-circRNAs and the bidirectional interaction between target miRNAs and DEGs were predicted. Functional analysis was performed, and the protein-protein interaction (PPI) network and the circRNA-miRNA-mRNA network were established. A total of 456 DEGs and 2 DE-circRNAs were identified with 3 mRNA expression profiles and 2 circRNA expression profiles. GO analysis indicated that DEGs were mainly enriched in extracellular matrix and cell adhesion, and KEGG confirmed that DEGs were mainly associated with focal adhesion, the PI3K-Akt signaling pathway, extracellular matrix- (ECM)- receptor interaction, and gastric acid secretion. 15 hub DEGs (BGN, COL1A1, COL1A2, FBN1, FN1, SPARC, SPP1, TIMP1, UBE2C, CCNB1, CD44, CXCL8, COL3A1, COL5A2, and THBS1) were identified from the PPI network. Furthermore, the survival analysis indicate that GC patients with a high expression of the following 9 hub DEGs, namely, BGN, COL1A1, COL1A2, FBN1, FN1, SPARC, SPP1, TIMP1, and UBE2C, had significantly worse overall survival. The circRNA-miRNA-mRNA network was constructed based on 1 circRNA, 15 miRNAs, and 45 DEGs. In addition, the 45 DEGs included 5 hub DEGs. These results suggested that hub DEGs and circRNAs could be implicated in the pathogenesis and development of GC. Our findings provide novel evidence on the circRNA-miRNA-mRNA network and lay the foundation for future research of circRNAs in GC.

## 1. Introduction

Gastric cancer (GC) is one of the most common malignancies worldwide, with the morbidity and mortality of GC ranking second among all cancers, especially in East Asia, Eastern Europe, and South America [1]. Although much progress has been made in the diagnosis and treatment of GC, the 5-year overall survival rate remains poor (approximately 20-25%) [2]. Therefore, there is an urgent need to study the mechanism underlying the occurrence and development of GC in order to achieve early diagnosis, effective treatment, and good prognosis for GC.

Bioinformatics analysis, including the use of microarray expression data sets, protein/gene-protein/gene interaction networks, and gene annotation, can be used to study cancer progression and identify potential therapeutic targets for development [3]. In addition, bioinformatics analysis methods can overcome the inconsistent results in the literature as a result of different sample sizes or microarray platforms in individual studies [4]. A large number of studies have used bioinformatics analysis to predict biomarkers for cancer treatments [5–8].

Circular RNA (circRNA) is a special type of endogenous noncoding RNA formed by reverse splicing of exon or intron cyclization. circRNAs exhibit stability, conservation, abundance, and expression-specific expression [9]. Recent studies have found that circRNAs are associated with many types of cancer, and several circRNAs have been identified as novel cancer biomarkers [10]. circRNAs are members of the competing endogenous RNA (ceRNA) family and act as regulators of miRNA activity; they can competitively inhibit the binding ability of miRNAs and their mRNA targets [11]. Although there are an increasing number of studies on circRNAs, the biological functions of most circRNAs remain unclear. To this end, we constructed a circRNA-miRNA-mRNA network as a means to further evaluate the roles of dysregulated circRNAs and mRNAs in GC.

In this study, we identified 456 differentially expressed genes (DEGs) between 119 human GC tissues and paracancer tissues by analyzing three sets of mRNA expression profiles in a public Gene Expression Omnibus (GEO) data set. Next, functional enrichment analysis and pathway enrichment analysis were performed to explore the roles of these DEGs. Then, a protein-protein interaction (PPI) network was constructed and 15 hub DEGs and three important modules in the network were identified. To assess the prognostic value of these hub DEGs, we also performed the Kaplan-Meier analysis. In addition, two differentially expressed circRNAs (DE-circRNAs) between GC tissues and paracancer tissues were identified by analyzing the two circRNA expression profiles. Finally, a circRNA-miRNA-mRNA network was successfully constructed.

## 2. Materials and Methods

### 2.1. Microarray Data Source

In order to identify DEGs, three mRNA expression profiles (GSE13911, GSE79973, and GSE118916) were downloaded from the NCBI GEO database (https://www.ncbi.nlm.nih.gov/geo/), including 63 GC tissues and 56 paracancer tissues. Similarly, two circRNA expression profiles (GSE83521 and GSE93541) were downloaded to identify DE-circRNAs, including nine GC samples and nine normal gastric samples. The detailed profiles are shown in Supplementary Table 1.

### 2.2. Identification of DEGs and DE-circRNAs

The GEO2R online analysis tool (https://www.ncbi.nlm.nih.gov/geo/geo2r/) was used to select DEGs and DE-circRNAs. The selection criteria were ∣logFC | >1.0 and adj. *p* value < 0.05, and the selected mRNAs and circRNAs were DEGs and DE-circRNAs. Next, Venny 2.1.0 (https://bioinfogp.cnb.csic.es/tools/venny/) was used to create a Venn diagram to find the intersection [12]. Moreover, the resulting DEGs were converted from a gene symbol to Entrez ID using the DAVID database (https://david.ncifcrf.gov/summary.jsp) [13].

### 2.3. Gene Ontology Analysis and KEGG Analysis

Gene ontology (GO) analysis is used to provide gene annotation terms [14], while the KEGG database is used for pathway enrichment analysis [15]. clusterProfiler V3.14.0 is an ontology-based R software package for statistical analysis and visualization of functional clusters of genomes or gene clusters [16]. In this study, the GO analysis and pathway enrichment analysis of mRNA were performed using the clusterProfiler package. In addition, a *p* value < 0.05 and a *q* value < 0.05 were chosen as the cutoff criteria for significant pathway terms.

### 2.4. PPI Network and circRNA-miRNA-mRNA Network Construction

The STRING database is a search tool for the retrieval of interacting genes or proteins (https://string-db.org), which can then be used to establish a PPI network [17]. 456 DEGs were imported into the STRING database; an interaction score > 0.4 [18] was used as the extraction cutoff standard for the PPI pair. Then, Cytoscape_3.7.1 (https://cytoscape.org) was used to visualize the PPI network [19]. The cytoHubba plug-in can be used to screen hub DEGs with the node degree. The MCODE Plug-in was used to filter important modules in the PPI network with a degree cutoff ≥ 2, node score cutoff = 0.2, *K* − core ≥ 2, and max.depth = 100 as the cutoff criteria [18]. In addition, the obtained circRNA-miRNA pairs and miRNA-mRNA pairs were combined to construct a circRNA-miRNA-mRNA network. The network was also visualized with Cytoscape.

### 2.5. Verification of Gene Expression

UALCAN (http://ualcan.path.uab.edu/) is a website for online analysis and mining of TCGA databases; it includes 34 normal stomach tissues and 415 GC tissues [20]. In this study, this database was used to verify the expression of hub DEGs in the PPI network. For this, *p* < 0.05 was considered statistically significant.

### 2.6. Survival Analysis

The Kaplan-Meier plotter (https://kmplot.com/analysis/) is an online database that evaluates the prognostic value of biomarkers in breast cancer, ovarian cancer, lung cancer, GC, and other cancers [21]. In this study, the Kaplan-Meier plotter database was used to assess the overall survival of hub DEGs among 1065 samples of GC. The hazard ratio (HR) and corresponding 95% confidence intervals were calculated. *p* < 0.05 was considered statistically significant.

### 2.7. Prediction of circRNA and miRNA Targets

The Circular RNA Interactome online tool (https://circinteractome.nia.nih.gov/) was used to predict target miRNAs for DE-circRNAs [22]. The mirDIP database (http://ophid.utoronto.ca/mirDIP/index_confirm.jsp) was used to predict the bidirectional relationship between target miRNAs and DEGs [23]. Ten of the 30 software programs in the mirDIP database were selected (DIANA, PITA, PicTar, RNAhybrid, RNA22, TargetScan, miRDB, microrna.org, miRBase, and miRcode). The criteria for selection were (1) three or more of the 10 software programs are included and (2) the top 5% of the confidence class genes are considered possible target genes. The circRNA-miRNA-mRNA network was then constructed using the corresponding circRNAs, miRNAs, and mRNAs.

## 3. Result

### 3.1. DEG Analyses

**Figure 1:**
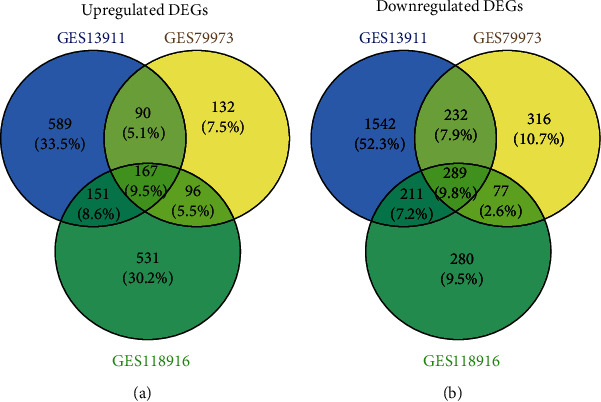
The identification of DEGs in the three data sets (GSE13911, GSE79973, and GSE118916). (a) Upregulated DEGs. (b) Downregulated DEGs. Different color regions represented different data sets. Overlapping areas are commonly DEGs. The cutoff criteria: ∣logFC | >1.0 and adj. *p* value < 0.05. DEGs: differentially expressed genes.

Three sets of mRNA expression profiles were obtained from the NCBI GEO database. Using the GEO2R online tool, 3271, 1399, and 1802 DEGs were extracted from the GSE13911, GSE79973, and GSE118916 data sets, respectively (∣logFC | >1.0 and adj. *p* value < 0.05). The extracted results were intersected and 456 DEGs were obtained, of which 167 were upregulated DEGs (Figure 1(a) and Supplementary Table 2) and 289 were downregulated DEGs (Figure 1(b) and Supplementary Table 2) in the GC tissues compared with the paracancer tissues.

### 3.2. GO and KEGG Pathway Enrichment Analyses of DEGs

**Figure 2:**
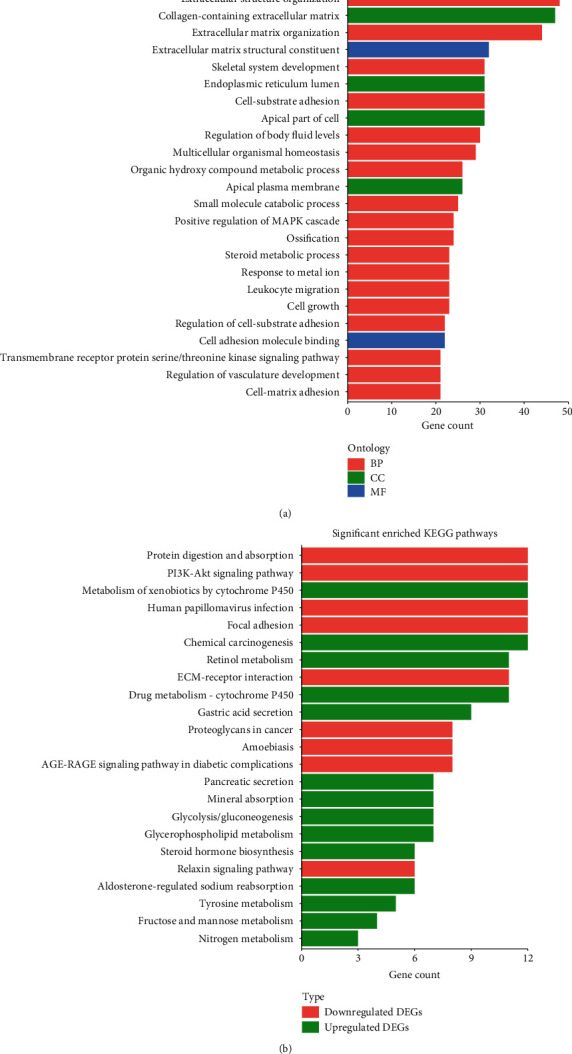
GO analysis and KEGG analysis of DEGs in GC. (a) The top 25 enriched GO terms of BP category, CC category, and MF category. (b) The significant enriched KEGG pathways.

The functions of the DEGs included three GO categories: molecular function (MF), biological process (BP), and cellular component (CC). The top 10 enriched GO terms showed that the DEGs were involved in an extracellular matrix, extracellular structure organization, collagen-containing extracellular matrix, extracellular matrix organization, extracellular matrix structural constituent, cell-substrate adhesion, skeletal system development, endoplasmic reticulum lumen, apical part of the cell, and regulation of body fluid levels (Figure 2(a)). The KEGG analysis confirmed that the DEGs were mainly associated with protein digestion and absorption, focal adhesion, the PI3K-Akt signaling pathway, extracellular matrix- (ECM-) receptor interaction, gastric acid secretion, and so on (Figure 2(b)).

### 3.3. PPI Network and Modular Analysis

**Figure 3:**
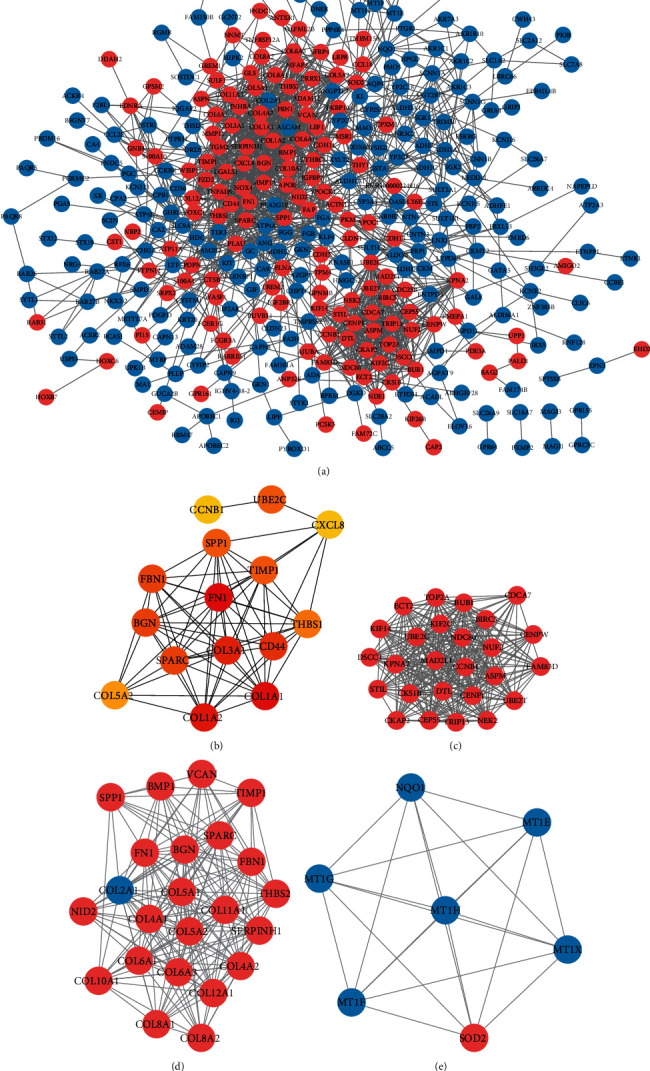
PPI network and modular analysis of DEGs. (a) The PPI network contains 369 nodes and 1570 edges; red represents upregulated DEGs, and blue represents downregulated DEGs. (b) The hub DEGs (degree: top 15) identified by the cytoHubba plug-in. The darker the color in the node, the higher the degree of interaction. (c) Module 1 contains 25 DEGs. (d) Module 2 contains 23 DEGs. (e) Module 3 contains 7 DEGs. The color of each node represents DEGs (red represents upregulated DEGs, and blue represents downregulated DEGs). PPI: protein-protein interaction.

456 DEGs were imported into the STRING database to explore the interrelationships between the various genes. 456 DEGs were used to establish the PPI network using the Cytoscape software. The PPI network consisted of 369 nodes and 1570 edges (Figure 3(a)). The cytoHubba plug-in in Cytoscape was used to screen the top 15 genes with node degree indicating the hub DEGs from the PPI network, including BGN, COL1A1, COL1A2, FBN1, FN1, SPARC, SPP1, TIMP1, UBE2C, CCNB1, CD44, CXCL8, COL3A1, COL5A2, and THBS1 (Figure 3(b) and Table 1). All were upregulated DEGs in the microarray expression profiles, and the mRNA expression levels of these genes were also significantly upregulated in GC tissues compared with normal stomach samples in the UALCAN database (Supplementary Figure 2). Based on the degree of importance, three key modules were then screened from the PPI network using the MCODE plug-in (Figures 3(c)–3(e)), and pathway enrichment analysis was performed. The results showed that Module 1 was mainly related to cell cycle, oocyte maturation, and platinum drug resistance (Supplementary Figure 1). Module 2 was primarily involved in the PI3K-Akt signaling pathway, ECM-receptor interaction, and focal adhesion (Supplementary Figure 1), while Module 3 was primarily involved in peroxisome, the FoxO signaling pathway, and so on (Supplementary Figure 1). In addition, the results indicated that 9 of the 15 hub DEGs (FN1, SPARC, FBN1, BGN, UBE2C, SPP1, TIMP1, COL5A2, and CCNB1) in the PPI network were distributed in the three modules, suggesting that these genes may have important roles in GC.

### 3.4. Survival Analysis of Hub DEGs

**Figure 4:**
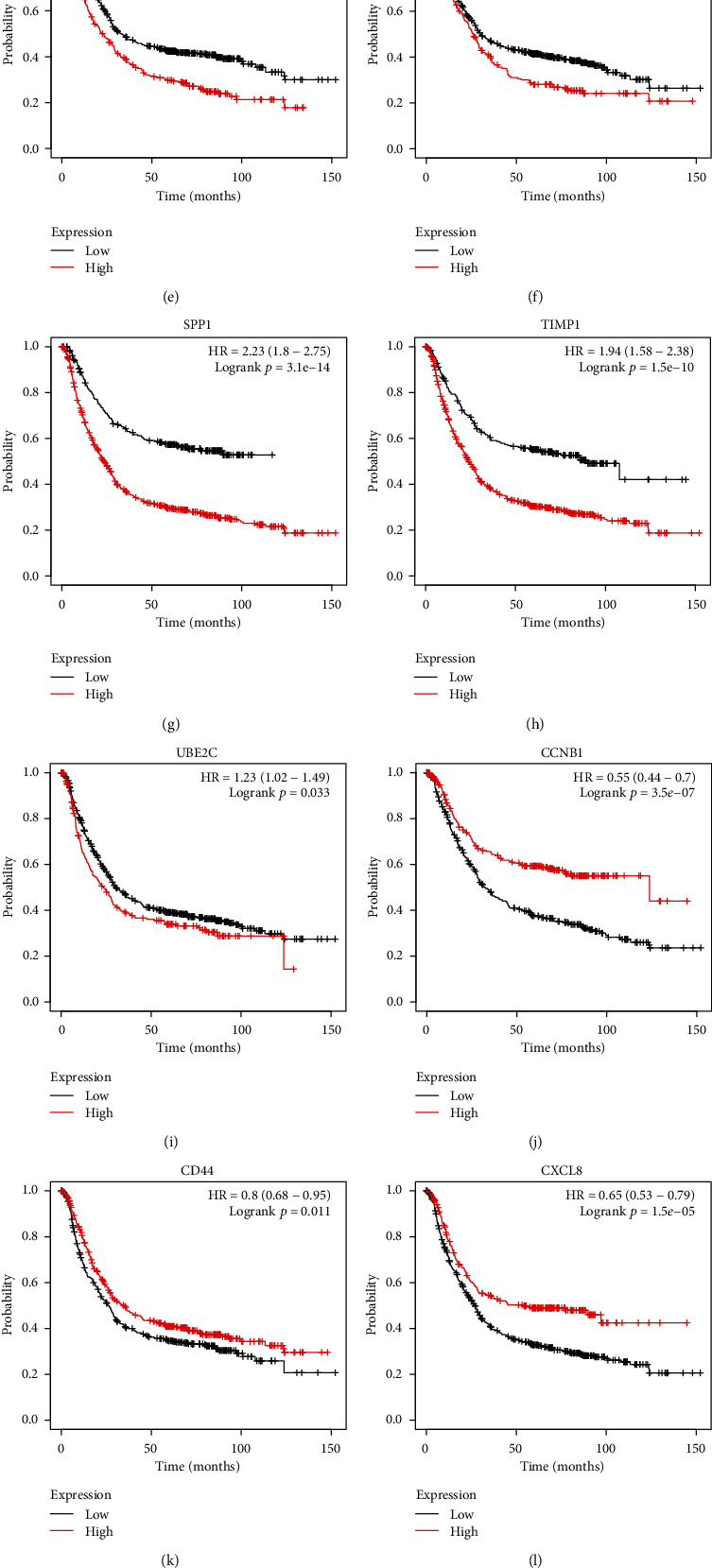
Survival analysis of the top 15 hub genes by the Kaplan-Meier plotter database in GC patient samples. (a–i) Survival analysis of BGN (a), COL1A1 (b), COL1A2 (c), FBN1 (d), FN1 (e), SPARC (f), SPP1 (g), TIMP1 (h), and UBE2C (i) by the Kaplan-Meier plotter database in GC patients. The results show that the survival of GC patients with high expressions of these DEGs was significantly worse (*p* < 0.01). (j–l) Survival analysis of CCNB1 (j), CD44 (k), and CXCL8 (l) by the Kaplan-Meier plotter database in GC patients. The data show that the survival of GC patients with high expressions of CCNB1, CD44, and CXCL8 were significantly better (*p* < 0.05). (m–o) Survival analysis of COL3A1 (m), COL5A2 (n), and THBS1 (o) by the Kaplan-Meier plotter database in GC patients. The result shows that COL3A1, COL5A2, and THBS1 were not associated with excessive survival in GC patients (*p* > 0.05).

The prognostic value of the 15 hub DEGs was assessed using the Kaplan-Meier plotter database. The results showed that nine hub DEGs (BGN, COL1A1, COL1A2, FBN1, FN1, SPARC, SPP1, TIMP1, and UBE2C) were associated with poor prognosis in GC patients (*p* < 0.05) (Figures 4(a)–4(i)). Furthermore, GC patients with high expression of CCNB1, CD44, or CXCL8 had a significantly more favorable prognosis (*p* < 0.05) (Figures 4(j)–4(l)). In addition, three hub DEGs (COL3A1, COL5A2, and THBS1) had nonsignificant logrank *p* values in GC patients (*p* > 0.05) (Figures 4(m)–4(o)).

### 3.5. DE-circRNA Analyses

**Figure 5:**
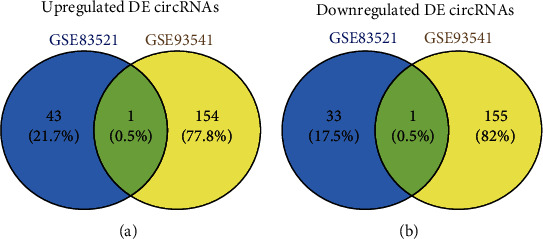
The identification of DE-circRNAs in the two data sets (GSE83521 and GSE93541). (a) Upregulated DE-circRNAs. (b) Downregulated DE-circRNAs. Different color regions represent different data sets. Overlapping areas are commonly DE-circRNAs. The cutoff criteria: ∣logFC | >1.0 and adj. *p* value < 0.05. DE-circRNAs: differentially expressed circRNAs.

The expression profiles of the two sets of circRNAs were obtained from the GEO database, including nine GC samples and nine normal gastric samples. Using the GEO2R online tool, 78 and 311 DE-circRNAs were extracted from the GSE83521 and GSE93541 data sets, respectively (∣logFC | >1.0 and adj. *p* value < 0.05). The extracted results were intersected, and two DE-circRNAs were obtained, of which, one DE-circRNA (hsa_circ_0001013) was upregulated and one DE-circRNA (hsa_circ_0021087) was downregulated in both the expression profiles (Figure 5).

### 3.6. Construction of the circRNA-miRNA-mRNA Network

**Figure 6:**
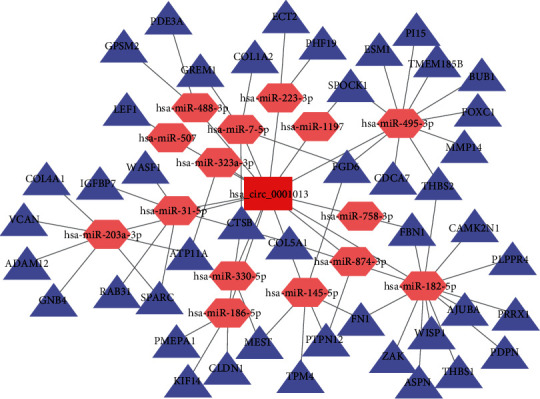
The circRNA-miRNA-mRNA network in GC. The circRNA-miRNA-mRNA network contains 61 nodes and 72 edges. The red rectangle represents DE-circRNA, the pink hexagons represent miRNAs, and the purple triangles represent DEGs.

To better understand the role of DE-circRNAs in GC, a circRNA-miRNA-mRNA network was established. Using the Circular RNA Interactome online tool, there were 108 target miRNAs for hsa_circ_0001013 and 11 target miRNAs for hsa_circ_0021087. We selected the bidirectional search function in the mirDIP database and then entered 167 upregulated DEGs and 108 miRNAs that were targeted by upregulated hsa_circ_0001013. Ten classic databases were selected to predict the bidirectional relationship between miRNAs and upregulated DEGs. The same method was used to predict the targeting relationship between 289 downregulated DEGs and 11 target miRNAs of downregulated hsa_circ_0021087. The results showed that 15 miRNAs targeted 45 upregulated DEGs and also interacted with hsa_circ_0001013 (Supplementary Table 3), while no miRNAs simultaneously targeted the downregulated DEGs and hsa_circ_0021087. The circRNA-miRNA-mRNA network of hsa_circ_0001013 was visualized using the Cytoscape software (Figure 6). The hsa_circ_0001013 network included 15 miRNAs and 45 DEGs, forming 57 pairs of circRNA/miRNA/mRNA axes. For instance, hsa_circ_0001013 is the ceRNA of miR-182-5p targeting FBN1, FN1, THBS1, AJUBA, ASPN, CAMK2N1, COL5A1, ZAK, PDPN, PLPPR4, PRRX1, THBS2, and WISP1. Furthermore, hsa_circ_0001013 is the ceRNA of hsa-miR-145-5p targeting FN1, FGD6, MEST, PTPN12, and TPM4. In addition, the 45 DEGs included the five hub DEGs of the PPI network: THBS1, FN1, FBN1, SPARC, and COL1A2. These RNA interactions may provide new insight into the mechanism underlying GC.

### 3.7. GO and KEGG Pathway Enrichment Analyses in circRNA-miRNA-mRNA Network

**Figure 7:**
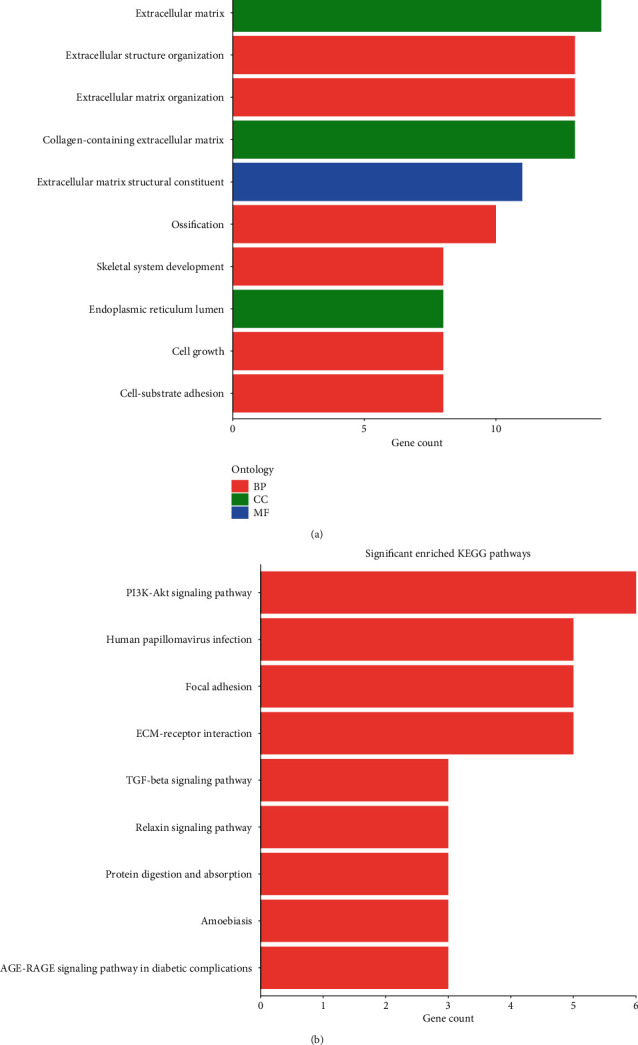
GO analysis and KEGG analysis of circRNA-miRNA-mRNA networks in GC. (a) GO analysis of the 45 DEGs in the circRNA-miRNA-mRNA networks. (b) KEGG pathway enrichment analysis of the 45 DEGs in the networks.

To date, the functions of hsa_circ_0001013 have not been reported. Therefore, a functional forecast of hsa_circ_0001013 was performed according to the mRNA annotations in the hsa_circ_0001013-related ceRNA network. GO analysis indicated that the function prediction of the circRNA-miRNA-mRNA network was mainly related to ECM and adhesion (Figure 7(a)). The KEGG analysis showed that the network was mainly enriched by the following terms: the PI3K-Akt signaling pathway, ECM-receptor interaction, focal adhesion, and human papillomavirus infection (Figure 7(b)).

## 4. Discussion

The tumorigenesis and development of GC is a relatively complex process because of the involvement of aberrations in gene expression regulatory networks. Traditionally, gene expression regulation analyses mainly focused on protein-coding genes (mRNAs), until the discovery of disease-related noncoding RNAs, including circRNAs. Still, little is known about the regulatory mechanisms of circRNAs in GC. To address this gap in understanding, this study used microarray data and performed bioinformatics analysis to identify disease-associated hub DEGs and the circRNA-miRNA-mRNA network.

This study obtained 456 DEGs based on three microarray profiles, including 167 upregulated genes and 289 downregulated genes. GO analysis showed that the 456 DEGs were mainly related to ECM, extracellular tissue, and collagen-containing ECM. Similarly, the results of KEGG analysis showed that the upregulated DEGs were not only enriched in protein digestion and absorption and the PI3K-Akt signaling pathway, but were also involved in focal adhesion and ECM-receptor interaction. The most significant pathway for downregulated DEGs was the metabolism of xenobiotics by cytochrome P450 (CYP3A5, CYP2C9, CYP2S1, ADH1A, ADH1C, ADH7, AKR1C1, AKR7A3, SULT2A1, UGT2B15, ALDH3A1, and GSTA1). Cytochrome P450 (CYP) is present in extrahepatic tissues and plays a key role in target tissue metabolic activation of xenobiotic compounds, the detoxification of toxic compounds following CYP-catalyzed biotransformation, and activation of inert xenobiotics, including drugs, to become toxicants [24]. A recent study found that low expression of ADH1A, ADH1C, and ADH7 was significantly associated with the increased risk of mortality for GC patients receiving 5-fluorouracil- (5-FU-) based adjuvant chemotherapy [25]. However, how the deficient CYP enzymes contribute to oncogenicity in the development of GC is not yet clear. It is worth noting that both GO analysis and KEGG analysis revealed relationships with ECM. ECM is a key regulator of cell behavior and phenotype; it activates or suppresses distinct sets of intracellular signaling pathways to modulate downstream cellular decisions, such as focal adhesions and collagen remodeling [26]. In many different types of cancer, ECM is highly dysregulated and the loss of tissue ECM homeostasis and integrity is seen as a hallmark of cancer and the typical transitional events in progression and metastasis [27]. Our data showed that GC progression was powerfully influenced by ECM and ECM-related events. Thus, remodeling of ECM in GC patients should be considered a potential therapeutic target.

To explore the molecular mechanism of GC, a GC-related PPI network was constructed and 15 hub DEGs were identified. These hub DEGs were all overexpressed in GC tissues. The Kaplan-Meier plotter was used to evaluate the effects of the 15 hub genes on the survival of GC patients. The results showed that overexpression of BGN, COL1A1, COL1A2, FBN1, FN1, SPARC, SPP1, TIMP1, and UBE2C led to poor prognosis in GC. As the main components of ECM, SPARC (acid-secreted and cysteine-rich secreted protein), BGN (biglycan), FBN1 (fibrillin 1), SPP1 (secreted phosphoprotein 1), fibronectin (FN1), and collagen play important roles in cell proliferation, differentiation, migration, and metastasis in GC [28]. Typically, two COL1A1 chains pair with one COL1A2 chain to form a triple helix of type I collagen [29]. Studies have shown that COL1A1 and COL1A2 are more highly expressed in GC tissues than in normal tissues, and both are related to the invasion and proliferation of GC cells [30, 31]. Type I collagen is required for collagen in bones, but it is also involved in ECM synthesis and in the promotion of changes to cell shape. The SPARC protein product has been associated with a variety of cancers, including GC [32]; it may affect GC metastasis by regulating the tumor microenvironment [33]. FBN1 is an extracellular matrix glycoprotein that can be used as a structural component of calcium-bound microfibers. The expression of FBN1 is upregulated in GC tissues and cells; the silence of FBN1 can inhibit the proliferative, migratory, and invasive abilities of GC cells [34]. As a high molecular weight glycoprotein, recent evidence has shown that FN1 is associated with a variety of cancers, and it is also involved in the invasion and migration of GC [35]. BGN (biglycan) is a member of the leucine-rich small proteoglycan family; it can enhance the invasion, migration, and formation of endometrial cancer cells [36], and it can promote the invasion of GC cells by activating the FAK signaling pathway [37]. The protein encoded by SPP1, also known as osteopontin, facilitates the combination of mineralized bone matrix and osteoclasts and is overexpressed in a variety of cancers, including breast cancer, lung cancer, pancreatic cancer, and GC [38]. Overexpression of SPP1 leads to poor GC prognosis, and positive osteopontin has an important role in the prediction of blood-borne metastasis [39]. UBE2C encodes a member of the E2 ubiquitin-binding enzyme family, which is involved in the ubiquitin-proteasome system and plays important roles in cell mitotic withdrawal and cell cycle progression. Knocking down UBE2C can inhibit the occurrence and development of GC through the Wnt/*β*-catenin and PI3K/Akt signaling pathways [40]. UBE2C is also related to poor GC prognosis [41]. TIMP1, a member of the TIMP gene family, is a natural inhibitor of matrix metalloproteinase. TIMP1 plays important roles in cell proliferation, tumorigenesis, angiogenesis, and antiapoptosis [42]. It has been reported that TIMP1 can be overexpressed in human GC by relying on the NF-*κ*B pathway and has the ability to regulate the proliferation of GC cells [43].

The current results revealed that overexpression of CCNB1, CD44, and CXCL8 was associated with significantly improved prognosis of GC patients. Studies have confirmed that CD44 is a nonkinase transmembrane glycoprotein that can promote tumor cell proliferation and invasion [44]. CD44 is also the most specific biomarker for the detection and isolation of oncogenic and chemoresistive cancer stem cells in noncardiac GC [45]. CXCL8 is an important member of the CXC chemokine family and plays an important role in the proliferation, migration, and activation of inflammatory systems in tumor cells [46]. CCNB1 (encoding cyclin B1) plays a vital role in cell mitosis. Overexpression of CCNB1 contributes to the proliferation of colorectal cancer cells [47], promotes the proliferation, migration, and invasion of bladder cancer [48], and also leads to poor prognosis of hepatocellular carcinoma [49]. CCNB1 can promote the proliferation and metastasis of GC by participating in the composition of the heterogeneous ribonucleoprotein-CCNB1/CENPF axis [50]. The overexpression of CCNB1, CD44, and CXCL8 in this study significantly improved the quality of life of GC patients. Therefore, the biological significance of these three genes in GC needs to be determined in further research. Taken together, these findings indicate that these nine hub DEGs are closely related to the prognosis of GC patients. As such, they may be potential prognostic indicators in GC.

Recent studies have found that circRNAs are associated with many types of cancer, and circRNAs have been proposed as novel cancer biomarkers [10]. The biological function of circRNAs is a “miRNA sponge”; circRNAs can competitively inhibit the binding ability of miRNAs and their mRNA targets [11]. There are an increasing number of studies on the role of circRNAs in GC, but the biological functions of most circRNAs remain unclear. In this study, we identified one upregulated DE-circRNA (hsa_circ_0001013) and constructed a circRNA-miRNA-mRNA network to estimate the function of hsa_circ_0001013 in GC. The results showed that hsa_circ_0001013 might play pivotal regulating roles in the gene expression of ECM and adhesion, and it also appears to be involved in the PI3K-Akt signaling pathway and ECM-receptor interaction in GC. Although there is currently no relevant research on the function of hsa_circ_0001013, the network revealed some important information. For instance, it was found that miR-182-5p, as a suppressed miRNA, improves migration and invasion of GC [51]. In our data, hsa_circ_0001013 was found to regulate the expression of FBN1, FN1, THBS1, and so on, through competing miRNA response elements (MREs) of miRNA-182-5p, which results in adhesion and metastasis in GC. In addition to miR-182-5p, studies have also shown that miR-758-3p and miR-145-5p are also downregulated in GC tissues and play important roles in promoting the migration and invasion of GC cells [52, 53]. In our circRNA-miRNA-mRNA network, the hsa_circ_0001013/miR-145-5p/FN1 axis and the hsa_circ_0001013/miR-758-3p/FBN1 axis may also affect the development of GC. These data indicate that circRNA plays a key role in GC. It is worth noting that miR-1197, miR-323-3p, miR-507, and miR-330-5p have not been studied in GC, but studies have shown that they play key roles in other tumors. For example, downregulated miR-1197 can inhibit the progression of human non-small-cell lung cancer by upregulating HOXC11 [54]. Further, miR-323-3p can inhibit the apoptosis of human lung cancer cells and inhibit the invasion and metastasis of pancreatic ductal adenocarcinoma cells [55, 56]. Moreover, miR-507 participates in the hsa_circ_0005394/miR-507/E2F3 axis and affects the process of hepatocellular carcinoma [57]. miR-330-5p can affect the expression of ELK1 and thus affect the proliferation, migration, and invasion of colon cancer cells [58]. It can also affect the expression of KLK4 to change the development process of ovarian cancer [59]. The relationships in the circRNA-miRNA-mRNA network require further exploration. Nonetheless, this pioneering network might provide a novel understanding of GC. Further research on the associated functions of hsa_circ_0001013 is being carried out in our laboratory.

## 5. Conclusions

In summary, through bioinformatics analysis, we identified 15 hub DEGs, of which, nine hub DEGs were associated with poor prognosis of GC patients, including BGN, COL1A1, COL1A2, FBN1, FN1, SPARC, SPP1, TIMP1, and UBE2C. In addition, two DE-circRNAs were identified. In order to identify the regulatory mechanism of circRNAs in GC, we constructed a related circRNA-miRNA-mRNA regulatory network of hsa_circ_0001013. Although these results need to be further verified, these hub DEGs and previously unreported hsa_circ_0001013 may play crucial roles in GC and may provide new ideas for the diagnosis, prognosis, and therapeutic targeting of GC.

## Figures and Tables

**Table 1:** 15 hub DEGs in the PPI networks.

Gene symbol	Official full name	Degree	Expression in GC
FN1	Fibronectin 1	64	Upregulation
COL1A1	Collagen type I alpha 1 chain	45	Upregulation
COL1A2	Collagen type I alpha 2 chain	42	Upregulation
COL3A1	Collagen type III alpha 1 chain	41	Upregulation
CD44	CD44 molecule (Indian blood group)	40	Upregulation
SPARC	Secreted protein acidic and cysteine rich	36	Upregulation
FBN1	Fibrillin 1	36	Upregulation
BGN	Biglycan	36	Upregulation
UBE2C	Ubiquitin-conjugating enzyme E2 C	35	Upregulation
SPP1	Secreted phosphoprotein 1	35	Upregulation
TIMP1	TIMP metallopeptidase inhibitor 1	35	Upregulation
THBS1	Thrombospondin 1	34	Upregulation
COL5A2	Collagen type I alpha 2 chain	32	Upregulation
CXCL8	C-X-C motif chemokine ligand 8	31	Upregulation
CCNB1	Cyclin B1	31	Upregulation

## Data Availability

The data used to support the findings of this study are available from the corresponding author upon request.

## Supplementary Materials

Supplementary Materials We provide some supplementary materials to support this research. Supplementary figures include KEGG analysis of three significant modules in PPI (see Supplementary Figure 1) and validation of the expression of 15 hub DEGs in the TCGA database (see Supplementary Figure 2). Supplementary tables include expression profile information (see Supplementary Table 1), DEGs in all three gene expression profiles (see Supplementary Table 2), and information on the circRNA-miRNA-mRNA network (see Supplementary Table 3).
