# Supplementary figures and images for: Bioinformatics Analysis of Key Genes and circRNA-miRNA-mRNA Regulatory Network in Gastric Cancer
Source: Biomed Res Int. 2020 Aug 22;2020:2862701. doi: 10.1155/2020/2862701 (PMC7463386; doi:10.1155/2020/2862701)

**
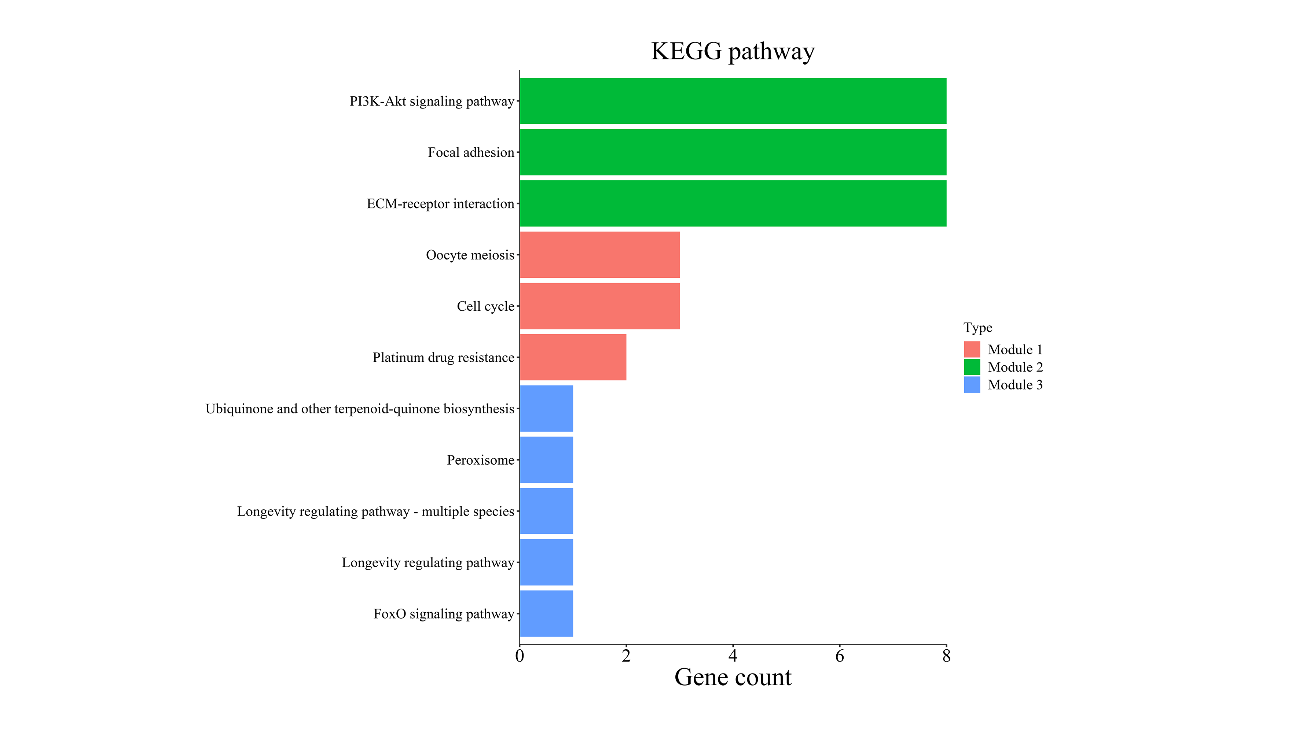
**

**Supplementary Figure 1:** KEGG analysis of three significant modules in PPI.

Supplement: Supplementary Materials — We provide some supplementary materials to support this research. Supplementary figures include KEGG analysis of three significant modules in PPI (see Supplementary Figure 1) and validation of the expression of 15 hub DEGs in the TCGA database (see Supplementary Figure 2). Supplementary tables include expression profile information (see Supplementary Table 1), DEGs in all three gene expression profiles (see Supplementary Table 2), and information on the circRNA-miRNA-mRNA network (see Supplementary Table 3). [file 2862701.f1.zip › Supplementary materials/Supplementary Figure 1.docx]
